# Efficient Cell Impedance Measurement by Dielectrophoretic Cell Accumulation and Evaluation of Chondrogenic Phenotypes

**DOI:** 10.3390/mi13060837

**Published:** 2022-05-27

**Authors:** Natsumi Nakata, Yuko Ishibashi, Shogo Miyata

**Affiliations:** 1 Department of Mechanical Engineering, Faculty of Science and Technology, Keio University, 3-14-1 Hiyoshi, Yokohama 223-8522, Japan; day.1380.n@gmail.com; 2 Graduate School of Science and Technology, Keio University, 3-14-1 Hiyoshi, Yokohama 223-8522, Japan; you.co.1021@gmail.com

**Keywords:** capacitance, chondrocyte, de-differentiation, dielectrophoresis, differentiation, electrical impedance, phenotype

## Abstract

The quantitative and functional analyses of cells are important for cell-based therapies. In this study, to establish the quantitative cell analysis method, we propose an impedance measurement method supported by dielectrophoretic cell accumulation. An impedance measurement and dielectrophoresis device was constructed using opposing comb-shaped electrodes. Using dielectrophoresis, cells were accumulated to form chain-like aggregates on the electrodes to improve the measurement sensitivity of the electrical impedance device. To validate the proposed method, the electrical impedance and capacitance of primary and de-differentiated chondrocytes were measured. As a result, the impedance of the chondrocytes decreased with an increase in the passage number, whereas the capacitance increased. Therefore, the impedance measurement method proposed in this study has the potential to identify chondrocyte phenotypes.

## 1. Introduction

The quantitative and functional analyses of living cells and tissues are important for the clinical application of cell therapies such as regenerative medicine. Although conventional analysis methods, including polymerase chain reaction, immunohistology, and flow cytometry using fluorescent dyes, are superior for evaluating cell types and phenotypes in detail, they require complex procedures and expensive instruments. Therefore, a simple, cost-effective, and quantitative evaluation method for cells and biological tissues is required.

Electrical impedance measurement is a promising approach for characterizing cell types, cell functions, and biological tissues. It has been used to determine simple cell properties, such as size [1] and concentration [2], as well as cell characteristics, such as cell phenotype, function, and viability [3,4,5,6]. Conventional analysis methods measure the impedance of a single cell or cell suspension using microscale electrodes and can characterize the properties of a single cell in detail [7,8]. However, these methods may not be appropriate for evaluating cell populations dispersed in buffer solution. Therefore, an efficient electrical impedance measurement device for cell populations such as cell suspensions is required for both clinical and research purposes. 

In this study, we hypothesized that the accumulation of cells on the electrodes to remove the buffer would improve the sensitivity of impedance measurements, even at lower cell concentrations in cell suspensions. Dielectrophoresis (DEP) is a promising approach for manipulating, patterning, and accumulating living cells [9,10,11,12,13]. It generates dipoles on a micro-particle in a non-uniform electric field [14]. An electrical force, called DEP force, is generated by the interaction between the dipoles and non-uniform electric field gradient. The direction of the DEP force is toward a higher electric field gradient (positive DEP) or repelled by a higher electrical field gradient (negative DEP), depending on the electrical properties of the cell and surrounding buffer. Previous studies reported cell patterning by positive and negative DEP forces [15,16,17,18]. Moreover, we previously reported some chondrocyte accumulation methods using both positive DEP [19] and negative DEP [20,21]. 

The purpose of this study is to establish an efficient impedance measurement method and improve its sensitivity by dielectrophoretic cell accumulation. We constructed transparent opposing comb-shaped electrodes on glass slides to enable both the DEP and impedance measurements of living cells. Using our proposed method, cells were accumulated on the electrodes by a positive DEP force, and electric impedance measurements were performed. Furthermore, the impedances of differentiated and de-differentiated chondrocytes were measured to verify the effectiveness of our method in identifying the differentiation and de-differentiation of chondrocytes. 

## 2. Materials and Methods

### 2.1. Experimental Design to Improve the Sensitivity of Electrical Imedance Measurement by Dielectrohpretic Cell Acccumulation

**Figure 1:**
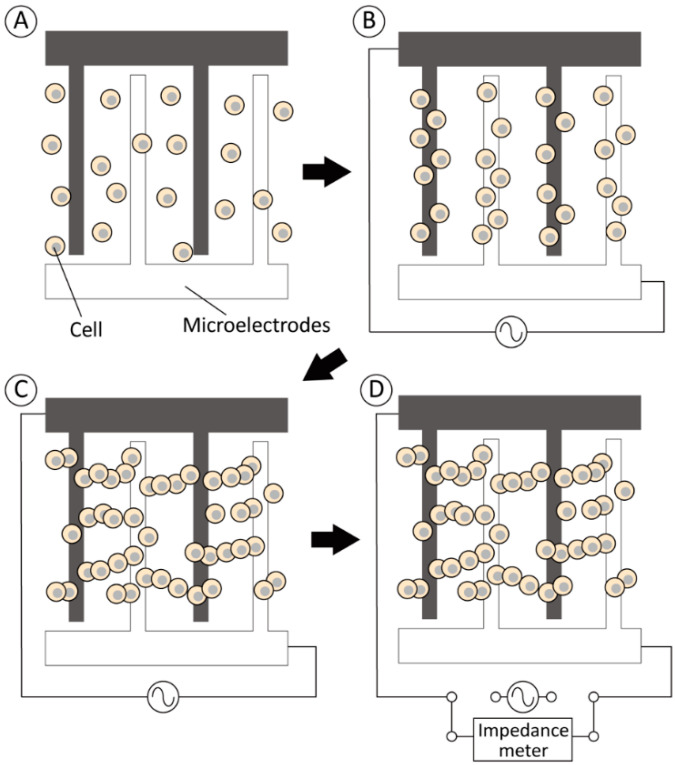
Experimental approach for improving the measurement sensitivity of cell electrical impedance by dielectrophoretic cell accumulation. Dispersed cells on opposing comb-shaped electrodes (**A**) were trapped on the electrodes by positive DEP forces (**B**) to form pearl-chain-like cell clusters (**C**). Finally, the electrical circuit was disconnected from the AC voltage source and (**D**) the cell impedance was measured by an impedance meter.

The efficient evaluation of the electrical impedance of living cells in cell suspensions requires a reduction in the effect of the surrounding buffer. In this study, positive DEP was used to form pearl-chain-like cell clusters [22]. In this case, cells in a non-uniform AC electric field generated by opposing comb-shaped electrodes were trapped on the electrodes to form pearl-chain-like cell clusters (Figure 1A–C). After the cell accumulation, the comb-shaped electrodes were disconnected from the AC voltage source and connected to an impedance meter to measure the electrical properties of the cells (Figure 1D). Our proposed method can increase the sensitivity of impedance measurements even under lower cell concentrations by cell accumulation and the formation of pearl-chain-like cell clusters on the microelectrodes. 

### 2.2. Chondrocyte Isolation and Culture with Multiple Passages

Primary and passaged calf articular chondrocytes were used for this study based on previous reports [23,24,25,26,27]. The chondrocytes were isolated from the shoulder joints of 3–5-week-old calves from a local abattoir [13,21]. Since all animals were slaughtered for food purposes, ethical permission was not required. Subsequently, cartilage explants were extracted from the humeral head of the shoulder joints and minced into 1-mm^3^ pieces. The minced tissues were then gently agitated in 0.2% collagenase type II digested with Dulbecco’s modified Eagle’s medium/Ham’s F12 (DMEM/F12) supplemented with 5% fetal bovine serum (FBS) and antibiotics–antimycotics for 12–16 h at 37 °C. The tissue-digested solution was filtered through a 70-μm nylon mesh filter (Cell Strainer, Corning, NY, USA) to remove the debris. Afterward, the chondrocytes were isolated from the cell-containing solution by centrifugation at 1500 rpm for 5 min and resuspended in phosphate-buffered saline. This procedure was repeated twice to wash the isolated chondrocytes. Finally, after centrifugation at 1500 rpm for 5 min, the chondrocytes were resuspended in a fresh culture medium (DMEM/F12 supplemented with 10% FBS and antibiotics–antimycotics) for cell culture. The cells were cultured on cell culture flasks to reach 80% confluence. After reaching the confluence, the cells were treated with 0.05% trypsin/EDTA and passaged in new flasks. The chondrocytes were cultured for multiple passages to induce de-differentiation. Based on PCR and immunohistology, previous studies reported that multiple passage induced de-differentiation of articular chondrocytes [25,26]. Primary calf chondrocyte could be passaged for 10 to 12 passages with maintaining proliferation. Primary chondrocytes (P0) and chondrocytes at different passage numbers (P1–P9) were prepared for impedance measurements. Before the experiments, the chondrocytes were suspended in a low-conductivity buffer (LC buffer; 10-mM HEPES, 0.1-mM CaCl_2_, and 59-mM D-glucose in sucrose solution) [12,19].

### 2.3. Electrical Impedance Measurement Device for Living Cells Supported by Dielectrohpretic Cell Accumulation

**Figure 2:**
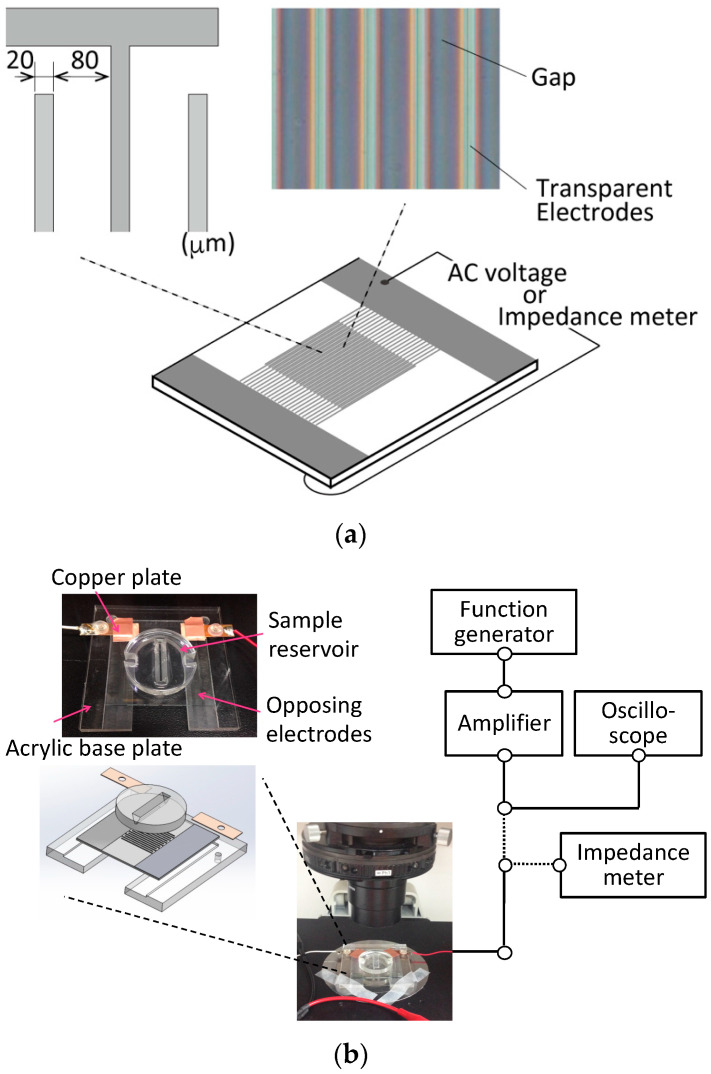
Experimental set-up for the electrical impedance measurement of a cell suspension. (**a**) Geometry of opposing comb-shaped electrodes: The width of each electrode line was 20 μm and the distance to the adjacent electrode was 80 μm. (**b**) Wiring diagram for the dielectrophoresis and impedance measurement system.

In this study, an impedance measurement device supported by DEP cell accumulation was developed. Both the impedance measurements and DEP were performed on opposing comb-shaped electrodes fabricated on a glass slide (Geomatec Co., Ltd., Yokohama, Japan) (Figure 2a). The electrodes were fabricated from glass slides coated with a conductive and transparent material, indium tin oxide. The width of each electrode line was 20 μm, and the distance to the adjacent electrode was 80 μm. For the cell suspension measurement, a liquid reservoir made of polydimethylsiloxane (PDMS) was set on the electrode-fabricated glass slide.

For DEP, an AC voltage was imposed by a function generator (WF1974, NF Corp., Yokohama, Japan), and an amplifier (BA4850, NF Corp., Yokohama, Japan) was connected to the electrodes (Figure 2b). The applied voltage was monitored using an oscilloscope (TDS1001B, Tektronix, Beaverton, OR, USA) connected in parallel to the amplifier. During DEP, the cells were moved in the direction of strong electric field gradients generated at the edge of the electrodes by positive DEP forces (Figure 1). After cell accumulation, the comb-shaped electrodes were disconnected from the AC voltage source and connected to an impedance meter (3532-80, Hioki Corp., Nagano, Japan) for electrical impedance and capacitance measurements. The impedance measurement and DEP device was set on a phase-contrast microscope (TE2000, Nikon, Tokyo, Japan) equipped with a digital camera to monitor the cell movement throughout the experiments.

**Figure 3:**
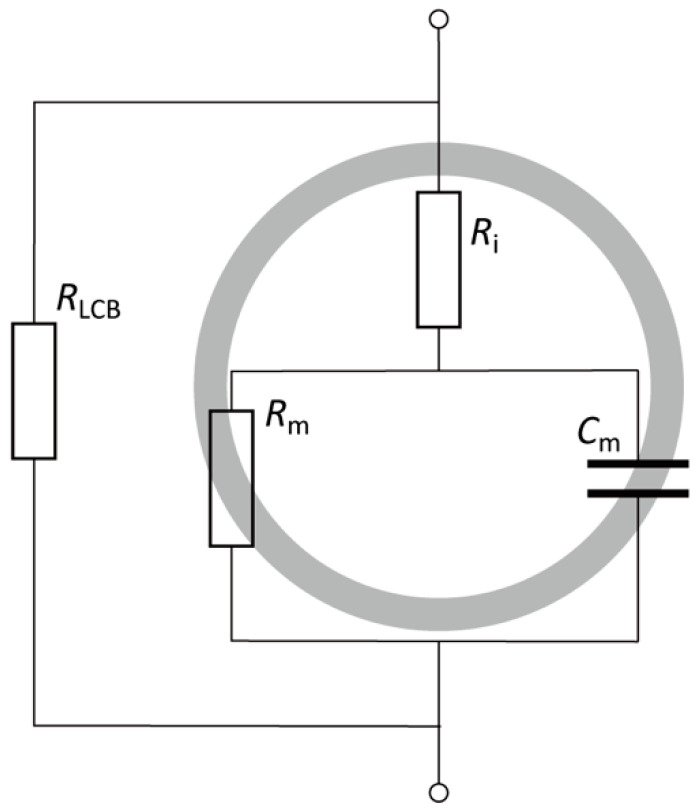
Equivalent circuit of the cell and surrounding buffer.

To evaluate the electrical properties of the cells, an equivalent circuit (Figure 3) was introduced to demonstrate the electrical conditions of the impedance measurement device [28]. The impedance of the cell suspension consists of the resistance of the LC buffer *R*_LCB_ and electrical components of the cells. Meanwhile, the electrical components of the cells are frequency dependent, consisting of the plasma membrane resistance *R*_m_ and capacitance *C*_m_ connected in parallel to each other and in series with the cytoplasm resistance *R*_cyto_. Additionally, the impedance of *C*_m_ and *R*_m_ in parallel is frequency dependent. Under ideal conditions, the cytomembrane capacitance dominates the impedance measurement and prevents electric current from penetrating the cytoplasm at lower frequencies. At higher frequencies, the plasma membrane acts as a high-pass filter for current to penetrate the cytoplasm. The measured impedance of the cell suspension exhibits the electrical properties of the plasma membrane and cytoplasm at lower and higher frequencies, respectively. Therefore, the electrical impedance |*Z*| and capacitance *C*_p_ were measured at 10 kHz, 100 kHz, and 1 MHz in this study.

### 2.4. Determination of the Experimental Conditions of the Electrical Impedance Measurement and Dielectrophoresis

To determine the appropriate cell concentration for the impedance measurement device, the electrical impedance measurements of cell suspensions with different cell concentrations were performed without DEP cell accumulation. As the representative of differentiated and de-differentiated chondrocytes, chondrocytes passaged twice (P2) were prepared and suspended in a LC buffer at concentrations of 0.1, 1.0, and 10 × 10^6^ cells/mL. Afterward, the cell suspension (300 μL) was injected into the PDMS reservoir, and the electrical impedance and capacitance at 100 kHz were measured at 0, 60, 120, 240, 480, and 720 s after the injection of the cell suspension. 

To evaluate cell accumulation by the positive DEP force, impedance measurements were performed after cell accumulation for 0, 60, 120, and 180 s. The cells were sedimented in the reservoir for 720 s before the impedance measurements and DEP experiments. An AC voltage of 10 V_p–p_ at 1 MHz was applied to cause positive DEP on the chondrocytes [21], and the electrical impedance and capacitance were measured at 100 kHz.

### 2.5. Characterization of the Relationship between Electrical Impedance and the De-Differentiation Process of Chondrocytes

The electrical impedance and capacitance of differentiated and de-differentiated chondrocytes were measured to verify the effectiveness of the proposed method in identifying chondrocyte phenotypes. According to the previous studies [26], de-differentiation of calf chondrocytes after passage 4 was confirmed by gene expression analyses. Primary chondrocytes and passaged cells (P1–P5, P9) were prepared and suspended in a LC buffer at 1.0 of 10^6^ cells/mL. The cell suspension (300 μL) was injected into the PDMS reservoir, and the cells were sedimented for 720 s before impedance measurements. The electrical impedance and capacitance were measured at 10 kHz, 100 kHz, and 1 MHz immediately after cell accumulation by positive DEP at a voltage of 10 V_p–p_ at 1 MHz for 180 s.

### 2.6. Statistical Analysis

Most of the data are representative of three individual experiments from different calf donors with similar results. For each experimental group, five to seven samples (*n* = 5–7) were analyzed, and each data point represents the mean and standard deviation. The statistical significance of the experimental data was evaluated using Bonferroni’s test.

## 3. Results and Discussions

### 3.1. Effect of Cell Concentration on Impedance Measurement

**Figure 4:**
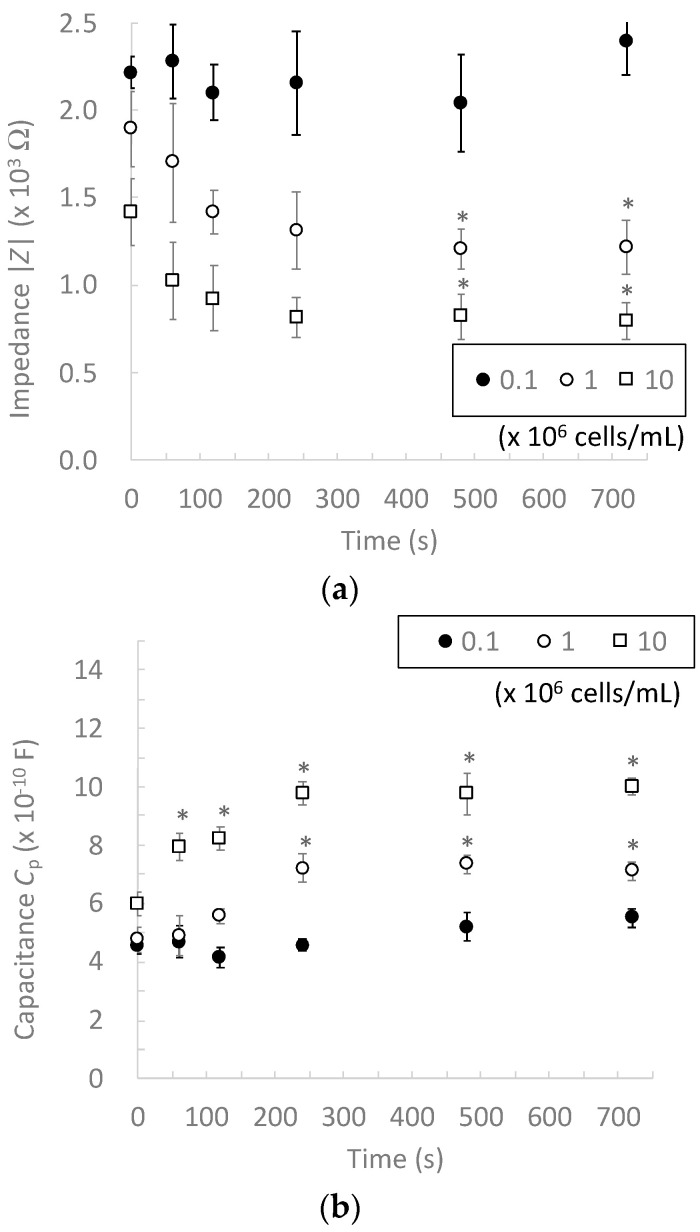
Changes in (**a**) electrical impedance and (**b**) capacitance of chondrocytes according to the cell sedimentation time at different cell concentrations (0.1, 1.0, and 10 × 10^6^ cells/mL). Mean +/− SD, *n* = 7. * indicates a significant difference in each value compared to that at 0 s, *p* < 0.05.

To determine the appropriate cell concentration for our impedance measurement device, the electrical impedances of 0.1, 1.0, and 10 × 10^6^ cells/mL chondrocyte suspensions were measured. The chondrocytes sedimented on the micro-electrodes according to the experimental time and impedance of 1.0 and 10 × 10^6^ cells/mL chondrocyte suspensions decreased with increasing experimental time and reached a plateau after 480 s, whereas that of the 0.1 × 10^6^ cells/mL suspension slightly changed during the experimental period and had a similar value to that of the LC buffer without chondrocytes (Figure 4). In this study, we used a LC buffer whose impedance was higher than that of the chondrocytes to contrast the difference between the electrical properties of chondrocytes and the surrounding buffer [13,20,21]. Therefore, in the chondrocyte suspension with a higher cell concentration, chondrocytes sedimented on the microelectrodes to decrease the electrical impedance. In suspensions with lower cell concentrations, the number of sedimented chondrocytes was not sufficient to change the electrical impedance. These results demonstrate that our impedance measurement device can measure electrical impedance at cell concentrations above 1.0 × 10^6^ cells/mL without dielectrophoretic cell accumulation.

### 3.2. Effect of Dielectrophoretic Cell Accumulation on Impedance Measurement

**Figure 5:**
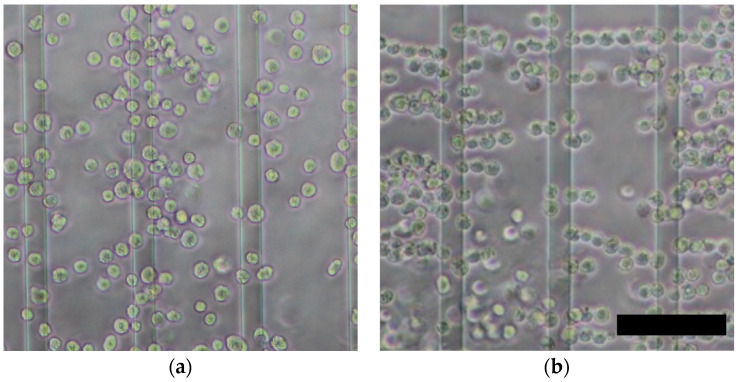
Formation of pearl-chain-like cell aggregates by positive DEP. Phase-contrast images of chondrocytes (**a**) before and (**b**) after positive DEP for 180 s. Scale bar = 100 μm.

**Figure 6:**
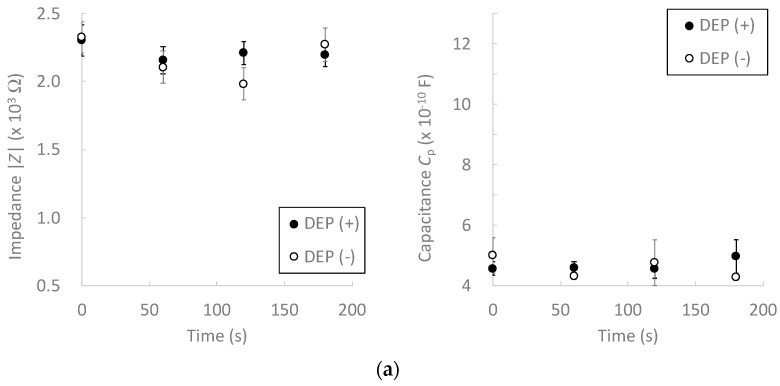

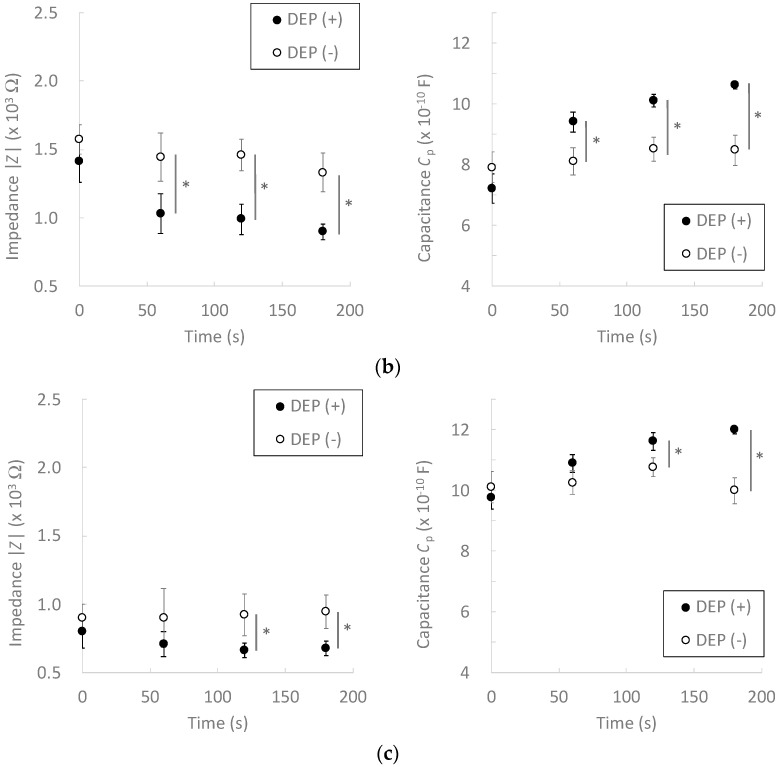
Effect of dielectrophoretic cell accumulation on the electrical impedance and capacitance of chondrocyte suspensions of (**a**) 0.1, (**b**) 1.0, and (**c**) 10 × 10^6^ cells/mL. Mean +/− SD, *n* = 7. * indicates a significant difference between DEP (+) and DEP (–) groups, *p* < 0.05.

Electrical impedance measurements were performed after cell accumulation by DEP force. During DEP, chondrocytes were accumulated on the microelectrodes and formed pearl-chain-like clusters, which bridged the adjacent electrodes (Figure 5). We previously reported that positive DEP forces were generated on bovine chondrocytes at 1 MHz of an AC electric field [21]. These phenomena are caused by the interaction between dipoles in the cells and the surrounding buffer [14]. At lower cell concentrations, neither the electrical impedance nor the capacitance changed significantly (Figure 6a). The electrical impedance decreased with an increase in the cell accumulation time, whereas the capacitance increased at higher frequencies (Figure 6b,c). After 120 s of cell accumulation, both the impedance and capacitance values reached a plateau. Furthermore, the standard deviation of the measured values in the DEP (+) condition tended to decrease with an increase in the DEP accumulation time. These results suggest that DEP cell accumulation can improve the measurement sensitivity and reduce the effect of the buffer near the microelectrodes. 

### 3.3. Relationship between Electrical Impedance and the Phenotypes of Chondrocytes

**Figure 7:**
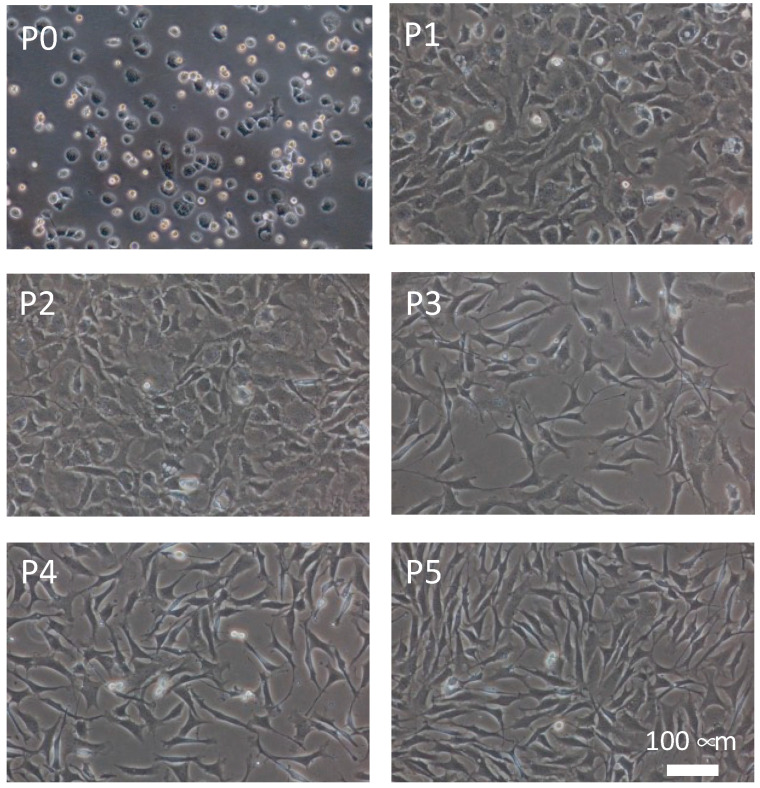
Phase-contrast microscopic images of primary chondrocytes (P0) and de-differentiated chondrocytes passaged for up to five times (P1–P5).

To verify the possibility of evaluating the de-differentiation phenotype of chondrocytes, electrical impedance measurements were performed on chondrocytes at different passage numbers. The chondrocytes were de-differentiated in an in vitro conventional monolayer culture (Figure 7). Primary chondrocytes showed a round shape, whereas passaged chondrocytes gradually became spindle-like in shape according to the passage number. The passaged chondrocytes showed these morphological changes with the de-differentiation of the chondrocyte phenotype [29]. In previous studies, de-differentiation of passaged chondrocytes was also confirmed by PCR and immunohistology [25,26]. It was considered that the chondrocytes gradually de-differentiated with an increase in passage number.

**Figure 8:**
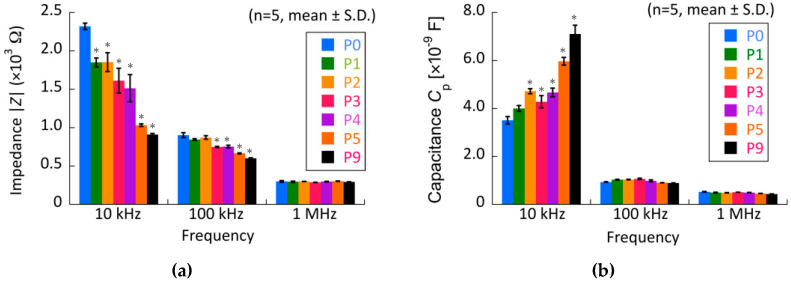
Effect of passage number on the (**a**) electrical impedance and (**b**) capacitance of chondrocytes. * indicates a significant difference in each value compared to that of primary cells (P0), *p* < 0.05.

The electrical impedance of the chondrocytes decreased with increasing passage number at 10 kHz, whereas the capacitance increased (Figure 8). The impedance and capacitance at 100 kHz and 1 MHz did not change dynamically with the passage number. Our experimental results suggest that impedance and capacitance at lower frequencies are related to chondrocyte morphology and function, such as differentiated and de-differentiated phenotypes. At lower frequencies, electric current could not penetrate the living cells but flowed through the plasma membrane during electrical impedance measurement because the plasma membrane had a lower conductivity and higher permittivity than the cytoplasm. Therefore, it was considered that the difference in the electrical impedance and capacitance of the passaged chondrocytes was related to structural changes in the plasma membrane. The cell size and ratio of cytoplasm volume to plasma membrane area should be evaluated to determine the structural change in the passaged chondrocytes. 

Wozniak et al. reported that the elastic modulus of chondrocyte plasma membranes decreased with an increase in passage number [30]. Furthermore, Sliogeryte et al. reported a relationship between the stiffness of de-differentiated chondrocytes and the strengthening of the plasma membrane by F-actin [31]. From these previous reports, it was considered that structural changes accompanied by ion channels and membrane proteins in the plasma membrane of chondrocytes are caused by cell de-differentiation. The mRNA expression or immunohistological analysis should be performed to evaluate the de-differentiation of chondrocytes and relationships with the electrical properties. To validate our method, a validation study using human cells should be also performed. However, based on our results, monitoring changes in electrical impedance and capacitance has the potential to assess phenotypic changes in de-differentiated chondrocytes.

## 4. Conclusions

In this study, we proposed electrical impedance measurements for living cells supported by DEP cell accumulation. To verify the proposed method, we developed an impedance measurement and DEP device using transparent opposing comb-shaped electrodes. Appropriate cell concentrations and DEP conditions were determined using bovine chondrocytes. Based on the results of this study, the measurement sensitivity of the device was improved by DEP cell accumulation. Furthermore, differentiated and de-differentiated chondrocytes were identified by measuring their electrical impedance and capacitance.
